# Using High-Definition Transcranial Alternating Current Stimulation to Treat Patients with Fibromyalgia: A Randomized Double-Blinded Controlled Study

**DOI:** 10.3390/life12091364

**Published:** 2022-08-31

**Authors:** Ashleigh Peng Lin, Chun-Chieh Chiu, Shih-Ching Chen, Yi-Jing Huang, Chien-Hung Lai, Jiunn-Horng Kang

**Affiliations:** 1 Department of Physical Medicine and Rehabilitation, Taipei Medical University Hospital, Taipei 110, Taiwan; 2 Department of Physical Medicine and Rehabilitation, School of Medicine, College of Medicine, Taipei Medical University, Taipei 110, Taiwan; 3 Department of Physical Medicine and Rehabilitation, Wan Fang Hospital, Taipei Medical University, Taipei 110, Taiwan; 4 School of Occupational Therapy, College of Medicine, National Taiwan University, Taipei 10051, Taiwan; 5 Graduate Institute of Nanomedicine and Medical Engineering, College of Biomedical Engineering, Taipei Medical University, Taipei 11031, Taiwan

**Keywords:** fibromyalgia, neuromodulation, transcranial stimulation, pain, quality of life, high-definition

## Abstract

Objectives: This study aimed to investigate the safety and efficacy of high-definition transcranial alternating current stimulation (HD-tACS) to the left primary motor cortex (M1) in the treatment of fibromyalgia (FM) patients. Methods: In this randomized, double-blind, sham-controlled clinical trial, patients with FM were recruited in a teaching hospital. Thirty-eight patients were randomized to active HD-tACS (*n* = 19) or sham stimulation (*n* = 19). Active stimulation included a daily session of 20-min stimulation of 1 mA HD-tACS over the left M1 for ten sessions in two weeks. The primary outcome was the change in pain intensity and quality of life, assessed using the numeric rating scale (NRS) and the fibromyalgia impact questionnaire (FIQ) at baseline and after two weeks of treatment. Secondary outcomes included other core symptoms of FM (psychological distress, sleep quality, hyperalgesia measured by pressure pain threshold) and changes in biomarkers’ total Tau and Aβ1-42. All analyses were based on intention-to-treat for a significance level of *p* < 0.05. Results: Of the 38 randomized patients, 35 completed the study. After two weeks, HD-tACS induced a significant reduction in FIQ score post-treatment. However, there were no significant differences in NRS and FIQ scores compared to sham stimulation. Most adverse events were mild in severity. Nevertheless, one patient receiving HD-tACS attempted suicide during the trial. Conclusions: These results suggest that HD-tACS may effectively reduce pain, psychological distress, and symptom impacts in FM patients. However, we found no significant differences between the two groups. Future studies investigating HD-tACS in FM are warranted.

## 1. Introduction

Fibromyalgia (FM) is a common debilitating condition characterized by widespread chronic pain, fatigue, sleep disturbance, impaired cognition, and anxiety and depression [1]. For many patients, the persistent symptoms lead to frequent health care use and impaired quality of life [2]. Most medications are inadequate for managing these symptoms and are associated with significant adverse effects and low tolerability [3]. The number needed to treat of amitriptyline, serotonin-noradrenalin reuptake inhibitors, and pregabalin, which are used in treating FM, ranges from 3.5 to 9 to achieve 30% pain relief [4,5,6]. Responses to these medications in FM patients are still unsatisfactory. Nonpharmacological interventions, such as cognitive-behavioral therapy, acupuncture, hydrotherapy, and meditation, have also shown limited efficacy in treating FM [7].

Non-invasive brain stimulation (NIBS) techniques are potentially promising for managing chronic pain disorders, such as migraines [8,9]. Two primary forms of NIBS widely used clinically are repetitive transcranial magnetic stimulation (rTMS) and transcranial direct current stimulation (tDCS) [10]. rTMS, which alters the neural activity of cortical areas involved in pain processing has shown positive results in FM patients [10]. However, high costs and relative inconvenience limit the use of rTMS in clinical practice [10]. By contrast, tDCS has the potential to become a cost-effective and easy-to-apply modality and is generally well-tolerated. tDCS uses low-intensity electrical currents to modulate cortical excitability and is suitable for chronic pain conditions such as FM [10].

Several studies in FM patients suggest that tDCS over the primary motor cortex (M1) can reduce chronic pain levels [11,12,13,14]. However, conventional tDCS stimulates broad cortical regions with poor spatial precision. High-definition tDCS (HD-tDCS), which uses a special arrangement of electrodes, provides more precise current delivery to target functional cortical areas [15]. Additionally, theta-burst stimulation (TBS), a modulated rTMS stimulation protocol, has garnered research attention, with significant neuromodulation effects in patients with chronic pain [16]. TBS uses pulses with inner high frequency (50 Hz), delivered at 5 Hz continuously for 40 s or intermittently (2 s every 10 s) for a total of 200 s [17]. TBS has been proposed to increase the long-term potentiation/depression on cortical synapses compared to the conventional protocol [17]. Some studies have proposed that transcranial alternating current stimulation (tACS), a modification of the tDCS protocol, may achieve a similar effect to TBS [18]. During tACS, a weak alternating current is applied to the scalp to entrain neural oscillations at the stimulation frequency, which modifies their amplitude [19]. As pain is closely associated with abnormal neural oscillations, tACS may relieve pain by attenuating or resetting anomalous oscillatory brain activity [19].

There are limited studies of HD-tDCS or tACS on FM [20,21,22]. Therefore, we conducted a double-blinded, randomized controlled trial to investigate the efficacy of HD-tACS compared to sham stimulation in patients with FM. This study aimed to investigate the effectiveness of HD-tACS in reducing pain and improving the quality of life in patients with FM. We also aimed to explore the safety and tolerability of HD-tACS in FM patients.

## 2. Methods

We conducted a two-week, double-blind, randomized controlled trial at Taipei Medical University Hospital, Taipei, Taiwan. We recruited the participants from October 2020 to October 2021. The study was approved by the Institutional Review Board of Taipei Medical University (N202004140) and registered on clinicaltrials.gov (NTC04550598). All participants provided written informed consent before enrollment. We followed the Consolidated Standards of Reporting Trials (CONSORT) reporting guideline for randomized controlled trials.

### 2.1. Participants

Participants were recruited in the outpatient clinic of Taipei Medical University Hospital. Eligibility criteria were men and women at least 20 years of age with a formal diagnosis of FM made by a practicing physician according to the American College of Rheumatology 2016 FM diagnostic criteria [23]. Baseline perceptive pain levels were assessed using the 0–10 numeric rating scale (NRS), and patients were eligible if they had persistent pain higher than or equal to 4. Patients were excluded if they had (1) any transcranial stimulation contraindications, (2) had pacemakers, metallic implants, epilepsy, recent head trauma, stroke, meningitis, prior brain surgery, arrhythmia, bipolar disorder or schizophrenia, malignancy, or autoimmune disorders, (3) had medication changes within a week of starting the trial, (4) were pregnant, abusing drugs, or alcohol. Patients were allowed to continue taking their usual medication for their trial.

### 2.2. High-Definition Transcranial Alternating Current (HD-tACS) and Sham Stimulation

An independent statistician generated a stratified random number sequence for participants. Eligible patients were blocked and randomized to either sham or active HD-tACS with allocation concealment and remained blind to their treatment condition throughout the study.

**Figure 1:**
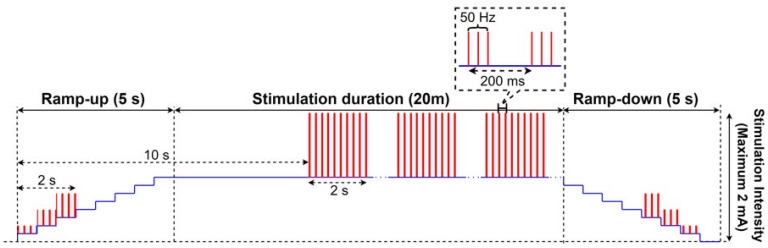
Alternative electrical stimulation was delivered as a monophasic square wave with a pulse width of 0.5 ms and intensity of 1 mA at 50 Hz, repeated at duty cycle with on time of 2 s and off time of 8 s. Sham HD-tACS consisted of a 10-s ramp-up period followed by 19 min and 40 s of no current stimulation, terminated by a 10-s ramp-up and ramp-down period.

HD-tACS was administered using a 4 × 1 ring electrode configuration. We targeted the left M1 by placing the anodal electrode on the C3 position in the 10/20 system for the EEG electrode positions. We set four cathodal electrodes in a radius of approximately 7.5 cm from the anode (corresponding to Cz, F3, T7, and P3 on the left hemisphere). The stimulation duration was 20 min. Alternative electrical stimulation was delivered as a monophasic square wave with a pulse width of 0.5 ms and intensity of 1 mA at 50 Hz, repeated at duty cycle with on time of 2 s and off time of 8 s (Figure 1). Sham HD-tACS consisted of a 10-s ramp-up period followed by 19 min and 40 s of no current stimulation that was terminated by a 10-s ramp-up and ramp-down period. The sham condition mimicked the skin sensation of active HD-tACS with insufficient duration to induce changes in cortical excitability. Participants received daily treatments for a total of 20 treatments in two weeks.

### 2.3. Assessments

Sociodemographic information was recorded. Participants were assessed by independent evaluators blinded to treatment conditions. The study’s primary outcome was the change in pain levels, assessed via the NRS, and the change in the quality of life, evaluated by the Fibromyalgia Impact Questionnaire (FIQ). Secondary outcomes were the changes of associated symptoms from baseline to endpoint. They included assessments of anxiety (Beck Anxiety Inventory, BAI) and depression (Beck Depression Inventory Second Edition, BDI-II), sleep quality (Pittsburgh Sleep Quality Index, PSQI), the pressure pain threshold (PPT), and biomarkers T-Tau and beta-amyloid 1–42 (Aβ1-42) proteins. These parameters were measured at baseline and after the final treatment.

### 2.4. NRS for Pain Assessment

Patients were asked to rate their current overall level of pain on a scale from 0 to 10 divided at 1-point intervals, with 0 being “complete absence of pain” and 10 “the worst pain imaginable.”

### 2.5. FIQ for Quality of Life

The FIQ assesses the impact and quality of life in patients with FM. It consists of 10 questions evaluating activities of daily living and pain and is divided into three main categories: function, overall impact, and symptoms. The maximum score is 100. A higher score indicates a more significant impact of the syndrome on the person.

### 2.6. BAI

BAI is a 21-item questionnaire measuring the severity of anxiety. The score for each item ranges from 0 to 3, with scores of 8 to 15 indicating mild, 16 to 25 indicating moderate, and greater than 26 indicating severe anxiety.

### 2.7. BDI-II

BDI-II evaluates the degree of depression and consists of 21 items measuring emotional, behavioral, and somatic symptoms. The score for each item ranges from 0 to 3, with total scores of 10 to 18 indicating mild, 19 to 29 indicating moderate, and greater than 30 indicating severe depression.

### 2.8. PPTs

PPT measures the minimum force applied to induce pain. PPTs were evaluated via delivery of direct pressure over four paired points of the body using a hand-held pressure gauge (Algometer, Pain Test, Wagner Inc., Greenwich, CT, USA). During measurement, the patient was in a relaxed sitting position. The pressure was gradually increased at a rate of approximately 2 lb/s until the patient experienced a transition from pressure to pain or a maximum of 25 lb. We measured PPTs in the areas of the trapezius, lateral epicondyle of humerus, greater trochanter, and knees three times, and averaged the results.

### 2.9. Plasma Biomarkers: T-Tau and Beta-Amyloid 1–42

Τ-Tau and Aβ1-42 are biomarkers of sleep disturbance [24]. Additionally, we previously demonstrated an elevation of these proteins in FM patients compared to controls [25]. As such, we measured Τ-Tau and Aβ1-42 levels pre- and post-stimulation. Blood samples (10 mL) were collected from the participants’ forearm veins in the morning after fasting for eight hours. Protease was immediately added to the blood sample to minimize protein degradation (cOmplete™, EDTA-free Protease Inhibitor Cocktail. Sn: 04693132001, Roche, Basel, Switzerland).

We collected whole blood into EDTA-treated tubes. The sample was centrifugated immediately for 15 min at 2000× *g* using a refrigerated centrifuge. The plasma was then transferred to fresh 1.5-mL tubes (1 mL of plasma per tube) and stored at −80 °C. An immunomagnetic reduction (IMR) assay was used to measure the levels of T-Tau protein and Aβ1-42 in the blood. The antibodies to the Aβ1-42 and Tau conjugated on the surface of 50 nm-in-diameter Fe3O4 magnetic particles (MF-AB2-0060 and MF-TAU-0060, MagQu Co., Ltd. New Taipei City, Taiwan) were used. The magnetic particles can form clusters when the antibodies bind with the target protein. The alternating current susceptibility of magnetic particles can be detected (Xacpro-S, MagQu Co., Ltd. New Taipei City, Taiwan) to analyze the signal of reagents. For t-tau, the reagents (MF-TAU0060) and plasma samples were mixed at a ratio of 2:1. For Aβ1-42 peptides, a 1:1 ratio of reagents (MF-AB2-0060) to plasma samples was used. After mixing, each sample for which IMR signaled was analyzed.

### 2.10. Adverse Effects

Adverse effects were registered using a standard form after each session. The patients reported whether they had any headache, neck pain, scalp pain, tingling, itching, burning sensation, skin redness, sleepiness, trouble concentrating, acute mood change, and other adverse effects after the stimulation. The severity of any adverse events and the degree to which they were related to the stimulation were recorded.

### 2.11. Statistical Analysis

Statistical analyses were performed using SPSS version 21 (SPSS Inc., Chicago, IL, USA). We used a Mann–Whitney U test to compare the mean for continuous variables and a Pearson Chi-square test or Fischer exact test for categorical variables between two groups. We used paired *t*-test to compare the difference of measured variables between pre- and post- treatment Significance was set at *p* ≤ 0.05. Results were reported as mean (SD).

## 3. Results

**Figure 2:**
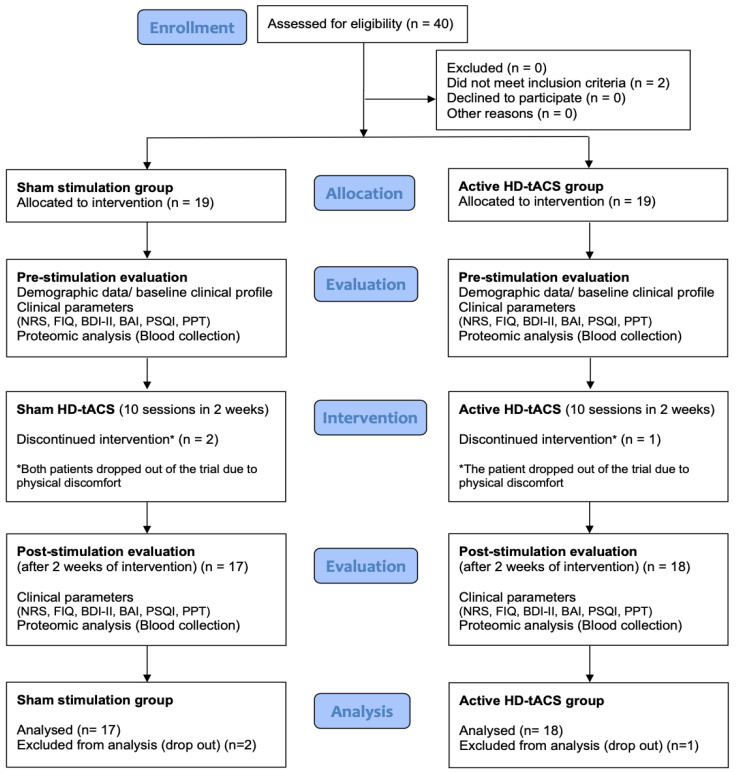
CONSORT flow chart of FM patients recruited in the trial. Among 40 patients, 2 were excluded for not meeting inclusion criteria. A total of 38 patients underwent randomization and 3 patients (2 in placebo and 1 in the experimental group) were lost to follow-up. A total of 35 patients, 17 in the sham stimulation group and 18 in the active HD-tACS group, completed the study. The primary outcomes, NRS and FIQ, and the secondary outcomes, BAI, BDI-II, PSQI, PPT, and biomarkers (tau and Aβ42), were assessed at baseline and the end of the study for statistical analysis.

The CONSORT flow diagram through the phases is illustrated in Figure 2. Of the 40 participants screened, two participants did not meet the inclusion criteria for FM and were excluded from the trial. Thirty-eight eligible participants were randomized to treatment (19 to sham and 19 to active stimulation). There were no demographic variables that differentiated the active and sham groups (mean [SD] age, 48.9 [12.3] for sham stimulation vs. 48.3 [13.6] for active stimulation; 17 women [89%] in the sham group vs. [68%] in the active stimulation group; Table 1). The sham and active stimulation groups both initially presented with FM (mean [SD] WPI, 10.7 [4.3] for sham stimulation vs. 9.4 [3.3] for active stimulation; mean [SD] SSS, 6.8 [2.5] for sham stimulation vs. 7.1 [3.2] for active stimulation). Both groups had comparable distributions of participants regarding baseline self-reported symptoms of depression, sleep quality, and biochemical profiles (Table 1).

BAI: Beck Anxiety Inventory; BDI-II: Beck Depression Inventory Second Edition; FIQ: Fibromyalgia Impact Questionnaire; HD-tACS: high-definition transcranial alternating current stimulation; NRS: numerical rating scale; PPT: pressure pain threshold; PSQI: Pittsburgh Sleep Quality Index; Tau: Tau protein; Aβ-42: Beta-amyloid protein 42 amino acid.

One participant receiving active stimulation and two receiving sham stimulation dropped out of the study. During the trial, the participant receiving active stimulation dropped out after a cardiopulmonary failure due to a suspected self-administered drug (beta-blocker) overdose. After an interview with a psychiatrist, an impulsive suicidal attempt was suspected. The patient recovered without any sequelae after management. The two participants receiving sham stimulation dropped out of the study due to physical discomfort during stimulation.

Paired *t*-test analysis of pre- and post-clinical profiles showed significant improvements in pain levels or quality of life in both the sham and active stimulation groups. In the sham stimulation group, we observed a decrease in pain symptoms (mean [SD] NRS, 5.4 [1.6] pre-intervention vs. 4.2 [2.4] post-intervention; mean [SD] PPT, 3.9 [1.4] pre-intervention vs. 3.5 [1.8] post-intervention). Similarly, in the active stimulation group, we found an improvement in FM symptoms (mean [SD] FIQ, 56.9 [16.3] pre-intervention vs. 48.7 [13.6] post-intervention). Self-reported depression and sleep quality symptoms also improved significantly in both groups (Table 2 and Table 3).

An independent sample non-parametric test comparing the pre-post difference, however, found no evidence of clinical superiority of active stimulation, as indicated by no significant difference in NRS and FIQ scores between the two groups (mean [SD] NRS, −1.1 [2.1] for sham stimulation vs. −0.5 [2.3] for active stimulation, *p* = 0.413; mean [SD] FIQ, −2.8 [23.6] for sham stimulation vs. −8.2 [11.9] for active stimulation, *p* = 0.400; Table 4). Similarly, there were no significant differences in any secondary outcomes between active and sham stimulation (Table 4). Changes in self-reported depressive symptoms (BDI and BAI) and sleep quality (PSQI) did not differ between two groups (Table 4). Biochemical profiles (T-Tau, ABeta1-42) were also not statistically significant between the two groups. T-Tau * ABeta1-42 was significantly higher in the active stimulation group (mean [SD], −30.2 [45.3] for sham stimulation vs. 8.5 [39.3] for active stimulation, *p* = 0.011).

Table 5 reports the distribution of adverse reactions between the active and sham stimulation. One participant receiving active stimulation had an acute cardiopulmonary failure. No other substantial adverse events occurred.

At the end of the trial, both active HD-tACS and sham stimulation had similar adverse reactions. Most of these adverse reactions were mild or moderate in severity. Nevertheless, one patient receiving active HD-tACS suffered from a cardio-pulmonary failure after a suicide attempt (medication overdose).

## 4. Discussion

In this double-blind sham-controlled trial, we investigated the efficacy of ten 20-min stimulation sessions of HD-tACS on the left M1 in improving pain intensity and associated core symptoms of FM. In 38 FM patients, we found that both active and sham stimulation reduced overall levels of perceived pain or improved quality of life. However, there was no significant difference between active and sham stimulation on the reported pain, anxiety and depression levels, and sleep quality. In general, the protocol used in this study proved well-tolerated. Nevertheless, one patient suffered from cardiopulmonary failure after an attempted suicide.

Anodal tDCS over M1 has been proposed as an effective intervention for managing clinical pain in FM [26]. FM is characterized by abnormal somatosensory processing and generalized central sensitization [27]. tDCS may modulate the sensory processing of pain in FM via modifications of the M1-thalamic inhibitory networks or other projections involved in pain processing [28] and has clinically been shown to improve pain perception [11,12,14,29]. In addition, several studies have found that tDCS over M1 is effective for other associated FM symptoms [30,31]. Conversely, other studies have found no significant differences in pain perception between active M1 tDCS and sham stimulation [32,33], with some attributing this deficiency to the poor spatial resolution of tDCS stimulation of cortical regions [15].

Previous studies have suggested that HD-tDCS provides improved pain response due to its precise stimulation of cortical targets [20]. A single session of HD-tDCS of the left M1 in FM patients led to significant reductions in overall perceived pain compared to sham [20]. Another study demonstrated that fifteen sessions of HD-tDCS could achieve 50% pain reduction in patients with FM but did not compare the results to sham stimulation [21]. Similarly, we found a 10% pain reduction after ten sessions of active HD-tACS stimulation. Nevertheless, we did not find significant differences in pain improvement after ten sessions of active M1 HD-tACS compared to sham stimulation. Moreover, when compared to sham, there were no superior treatment effects of active HD-tACS over M1 on other symptoms of FM, such as anxiety, depression, or sleep quality. One possible explanation for the non-significant results of NRS and PTT in the intervention group could be that tACS targets mainly the affective pain system, having a smaller effect on pain perception. In terms of the non-significant result of FIQ in the sham group, a possible explanation could be that the FIQ reflects a subjective overall functional status of patients. As such, patients in the sham group could have perceived an improvement in function despite having no active stimulation. Despite this discrepancy, our results are similar to other studies, which show pain improvements relative to baseline levels but nonsignificant differences between active and sham stimulation [11,34]. In addition, a meta-analysis of 27 studies showed heterogeneity and only a small effect size of active tDCS stimulation compared to sham (SMD −0.43, CI −0.63 to −0.22) for pain intensity [10].

We used tACS due to its ability to potentiate cortical excitability, which may induce more significant and longer-lasting changes than conventional methods [17]. Additionally, previous studies have shown the association between FM pain and abnormal neuronal oscillations, specifically general increases in theta, beta, and gamma waves along with decreases in the alpha peak [35]. tACS was intended to manipulate neural oscillation to restore normal thalamocortical rhythmicity in FM patients.

Our study complements previous tACS studies in the context of pain [22,36,37,38]. All four studies applied 5 or 10 Hz alpha stimulation targeting somatosensory areas. One study indicated an analgesic effect of tACS on the perceived pain intensity induced by brief experimental stimuli, but only when pain intensity was uncertain [38]. Another study in FM patients showed improved perceived pain intensity and cognitive symptoms with tACS coupled with physiotherapy, associated with increased alpha 1 (8–10 Hz) activity [22]. Similarly, a study in chronic back pain patients showed that tACS-induced changes in alpha activity correlated with pain intensity [36]. A study on healthy participants did not find significant tACS effects on reducing experimental tonic pain [37].

Although we expected better pain improvements with the tACS compared to sham, the data did not confirm any superiority of this protocol. One possibility is the differential matching of tACS frequencies with the intrinsic properties of sensorimotor networks involved in pain regulation [39]. It is essential to highlight that the optimal parameters of tACS still need to be defined. Previous studies demonstrate the limits of the tACS technique in inducing changes in cortical excitability [40]. Therefore, our results may indicate a ceiling effect of the cortical changes.

The present results may suggest a significant placebo response to the application of the sham protocol, which may have omitted the actual effects of active HD-tACS. Placebo analgesic effects can be brought about by the belief and expectation of symptom improvement and have been widely observed in neuromodulation studies for chronic pain [33,41]. Whether a placebo alters the transmission of sensory pain or is a product of the Hawthorne effect has garnered widespread debate [42]. Nonetheless, placebo stimulation activates the endogenous μ-opioid receptor in the periaqueductal grey matter, precuneus, and thalamus [43]. In addition, before active or sham stimulation, an early placebo effect has been shown to prime the endogenous μ-opioid receptor [44]. Therefore, the success of M1 tDCS analgesia could depend on the individual’s susceptibility to mobilize μ-opioid activity related to placebo. Moreover, as both tDCS and placebo are associated with the endorphin system, we cannot exclude the possibility of placebo-associated pre-activation of endorphin, which may have masked and saturated the analgesic effects of tDCS [30]. Potential strategies to overcome the placebo response include minimizing patient expectations and ensuring that the sham stimulation is indistinguishable from active stimulation. In addition, one could measure patients’ perception of the intervention and their expectations of treatment outcome early in the trial to test how their perception influences the outcome in the placebo and active groups [45].

Currently, no studies have shown significant effects of tDCS on blood metabolites in FM patients. Furthermore, there is currently a lack of FM-specific biomarkers in the blood [46]. As we previously found an elevation of serum T-Tau and Aβ1-42 levels in FM patients compared to controls, we sought to analyze these protein levels pre- and post-stimulation as objective indices of treatment response [25]. Given the short duration of the study, our stimulation paradigm expectedly did not translate into significant changes in the patient’s serum T-Tau or Aβ42 levels, which are both associated with long-term cognitive decline [47]. Long-term follow-up studies are needed to clarify the long-lasting cognitive effects of HD-tACS.

In the present study, one patient who received active stimulation attempted suicide with a drug overdose. Although no causality was established between this protocol and the attempted suicide, this incident prompted careful consideration of the effects of neuromodulation interventions on suicidal ideation and behavior. Although it is rare, antidepressant treatments have been shown to carry an increased risk of suicidal ideation in vulnerable patients [48]. Early activating effects of antidepressants may transiently increase suicide risk [49]. In addition, a study showed that theta tACS increased risk-related behavior [50]. Speculatively, neurostimulation may have a similar effect. The possibility of emergent adverse effects with this protocol cannot be overlooked. Further study should consider this potential risk when performing tDCS/tACS, particularly in patients with previous suicidal ideation/attempts. The majority of our tACS sessions proceeded uneventfully. They were not associated with any adverse effects other than a mild-to-moderate tingling or itching sensation, which in most participants subsided after the first few minutes of stimulation. These results support the generally good tolerability of this intervention in FM patients.

There are several insufficiencies in the present study. First, our study contained a small sample size. A large study is suggested to confirm our findings. Second, we did not perform follow-up assessments of patient pain levels and are unable to conclude long-term results of tACS on FM pain and associated symptoms. Third, the patients enrolled in the present study were Asians. Extrapolation of our results to other ethnic groups will need further investigation. Lastly, we did not have simultaneous EEG recordings to ensure that the tACS protocol could modulate neural oscillations, nor did we use fMRI to provide information on the distribution of the tACS current in the brain as a function of anatomy. Nevertheless, future studies may perform additional computational modeling to control the interindividual variability among FM patients.

## 5. Conclusions

We found that the FM patients showed significant improvement in pain, psychological distress, and symptom severity while receiving HD-tACS. However, no significant difference between pre- and post-treatment was noted between the patients receiving active and sham stimulation. The HD-tACS was generally well-tolerated during stimulation. Nevertheless, one patient conducted suicide unexpectedly in the present study. Caution on the potential linkage between neuromodulation and unexpected behaviors should be considered.

## Figures and Tables

**Table 1:** Baseline demographic and clinical characteristics of FM patients treated with active HD-tACS or sham stimulation.

Characteristic	Sham Stimulation (*n* = 19)	Active HD-tACS (*n* = 19)	*p*-Values *
Age, mean (S.D.), years	48.9 (12.3)	48.3 (13.6)	0.872
Gender, *n* (%)			0.118
Women	17 (89)	13 (68)	
Men	2 (11)	6 (32)	
Marital Status, *n* (%)			0.401
Single	7 (37)	8 (42)	
Married	10 (53)	10 (53)	
Divorced	0	1 (5)	
Widowed	2 (10)	0	
Clinical Profiles, mean (S.D.)			
WPI	10.7 (4.3)	9.4 (3.3)	0.250
SSS	6.8 (2.5)	7.1 (3.2)	0.821
NRS	5.3 (1.6)	5.0 (2.4)	0.636
FIQ	49.6 (16.6)	58.0 (16.5)	0.125
BDI-II	18.1 (11.8)	23.3 (11.2)	0.171
BAI	20.6 (11.4)	20.8 (10.5)	0.965
PSQI	11.5 (3.8)	12.1 (4.2)	0.662
PPT, kg/cm^2^	3.8 (1.5)	3.8 (1.6)	0.959

BAI: Beck Anxiety Inventory; BDI-II: Beck Depression Inventory Second Edition; FIQ: Fibromyalgia Impact Questionnaire; FM: fibromyalgia; HD-tACS: high-definition transcranial alternating current stimulation; NRS: numerical rating scale; PPT: pressure pain threshold; PSQI: Pittsburgh Sleep Quality Index; SSS: symptom severity scale; WPI: widespread pain index. * The mean difference is significant at the 0.05 level. (*p*-value < 0.05).

**Table 2:** Effects of sham stimulation on pain and clinical status. Pre- and post-sham stimulation pain and clinical parameters were evaluated by paired *t*-test analysis.

Outcomes	*n*	Mean	S.D.	Sig. (2-Tailed)
NRS (pre)	17	5.4	1.6	0.040 *
NRS (post)	17	4.2	2.4	
FIQ (pre)	17	49.5	16.5	0.629
FIQ (post)	17	46.7	28.5	
BDI (pre)	17	18.1	11.0	0.000 *
BDI (post)	17	12.5	9.4	
BAI (pre)	17	20.6	11.7	0.021 *
BAI (post)	17	16.4	11.0	
PSQI (pre)	17	11.8	3.9	0.025 *
PSQI (post)	17	9.9	4.0	
PPT (kg/cm^2^) (pre)	17	3.9	1.4	0.047 *
PPT (kg/cm^2^) (post)	17	3.5	1.8	
T-Tau (pg/mL) (pre)	17	23.6	3.5	0.090
T-Tau (pg/mL) (post)	17	22.3	3.4	
ABeta1-42 (pg/mL) (pre)	17	16.6	0.4	0.123
ABeta1-42 (pg/mL) (post)	17	16.6	0.4	
T-Tau*ABeta1-42 (pg/mL)^2^ (pre)	17	391.5	65.3	0.854
T-Tau*ABeta1-42(pg/mL)^2^ (post)	17	371.6	63.4	

BAI: Beck Anxiety Inventory; BDI-II: Beck Depression Inventory Second Edition; FIQ: Fibromyalgia Impact Questionnaire; *n*: number of subjects; NRS: numerical rating scale; PPT: pressure pain threshold; PSQI: Pittsburgh Sleep Quality Index; Tau: Tau protein; Aβ-42: amyloid-beta protein 42 amino-acid * significant at the 0.05 level (2-tailed).

**Table 3:** Effects of HD-tACS stimulation on pain and clinical status. Pre- and post-active HD-tACS stimulation pain and clinical parameters were evaluated by paired *t*-test analysis.

	*n*	Mean	S.D.	Sig. (2-Tailed)
NRS (pre)	18	4.9	2.4	0.376
NRS (post)	18	4.4	2.3	
FIQ (pre)	18	56.9	16.3	0.010 *
FIQ (post)	18	48.7	13.6	
BDI (pre)	18	22.8	11.3	0.002 *
BDI (post)	18	17.2	9.4	
BAI (pre)	18	20.4	10.7	0.003 *
BAI (post)	18	15.1	6.4	
PSQI (pre)	18	11.9	4.3	0.012 *
PSQI (post)	18	10.5	3.6	
PPT (kg/cm^2^) (pre)	18	3.8	1.6	0.375
PPT (kg/cm^2^) (post)	18	3.6	1.7	
T-Tau (pg/mL) (pre)	18	22.1	3.0	0.375
T-Tau (pg/mL) (post)	18	22.5	2.9	
ABeta1-42 (pg/mL) (pre)	18	16.5	0.3	0.968
ABeta1-42 (pg/mL) (post)	18	16.5	0.3	
T-Tau*ABeta1-42 (pg/mL)^2^ (pre)	18	365.3	52.6	0.375
T-Tau*ABeta1-42 (pg/mL)^2^ (post)	18	372.1	52.1	

BAI: Beck Anxiety Inventory; BDI-II: Beck Depression Inventory Second Edition; FIQ: Fibromyalgia Impact Questionnaire; *n*: number of subjects; NRS: numerical rating scale; PPT: pressure pain threshold; PSQI: Pittsburgh Sleep Quality Index; Tau: Tau protein; Aβ-42: amyloid-beta protein 42 amino-acid. * significant at the 0.05 level (2-tailed).

**Table 4:** Independent sample non-parametric test to compare the pre- and post-difference in sham and active HD-tACS stimulation.

	Group	*n*	Mean	S.D.	Sig. (2-Tailed)
NRS	Sham	17	−1.1	2.1	0.413
	Active HD-tACS	18	−0.5	2.3	
FIQ	Sham	17	−2.8	23.6	0.400
	Active HD-tACS	18	−8.2	11.9	
BDI	Sham	17	−5.6	5.0	0.963
	Active HD-tACS	18	−5.6	6.3	
BAI	Sham	17	−4.3	6.9	0.668
	Active HD-tACS	18	−5.3	6.5	
PSQI	Sham	17	−1.9	3.2	0.552
	Active HD-tACS	18	−1.4	2.1	
PPT (kg/cm^2^)	Sham	17	−0.3	0.7	0.527
	Active HD-tACS	18	−0.2	0.8	
T-Tau (pg/mL)	Sham	17	−1.2	2.8	0.068
	Active HD-tACS	18	0.4	2.3	
ABeta1-42 (pg/mL)	Sham	17	0.0	0.3	0.925
	Active HD-tACS	18	0.0	0.4	
T-Tau*ABeta1-42 (pg/mL)^2^	Sham	17	−30.2	45.3	0.011 *
	Active HD-tACS	18	8.5	39.3	

BAI: Beck Anxiety Inventory; BDI-II: Beck Depression Inventory Second Edition; FIQ: Fibromyalgia Impact Questionnaire; *n*: number of subjects; NRS: numerical rating scale; PPT: pressure pain threshold; PSQI: Pittsburgh Sleep Quality Index; Tau: Tau protein; Aβ-42: Beta-amyloid protein 42 amino acid. * significant at the 0.05 level (2-tailed).

**Table 5:** Adverse reactions in the HD-tACS and sham stimulation groups.

Adverse Reactions	Active HD-tACS (*n* = 18)	Sham (*n* = 17)
Headache	1 (5.6%)	0 (0.0%)
Neck pain	0 (0.0%)	1 (5.9%)
Scalp pain	2 (11.1%)	2 (11.8%)
Stinging	3 (16.7%)	1 (5.9%)
Itch	2 (11.1%)	2 (11.8%)
Burning sensation	1 (1.0%)	1 (5.9%)
Drowsiness	2 (11.1%)	1 (5.9%)
Difficulty Concentrating	1 (1.0%)	0 (0.0%)
Other	1 (5.6%) #	2 (11.8%)

HD-tACS: high-definition transcranial alternating current stimulation. # Cardio-pulmonary failure after a suicide attempt with medication overdose.

## Data Availability

Data available on request due to privacy/ethical restrictions.

## References

[1] Sarzi-Puttini P., Giorgi V., Marotto D., Atzeni F. (2020). Fibromyalgia: An update on clinical characteristics, aetiopathogenesis and treatment. Nat. Rev. Rheumatol..

[2] Sicras-Mainar A., Rejas J., Navarro R., Blanca M., Morcillo A., Larios R., Velasco S., Villarroya C. (2009). Treating patients with fibromyalgia in primary care settings under routine medical practice: A claim database cost and burden of illness study. Arthritis Res. Ther..

[3] Häuser W., Urrútia G., Tort S., Üçeyler N., Walitt B. (2018). Serotonin and noradrenaline reuptake inhibitors (SNRIs) for fibromyalgia. Cochrane Database Syst. Rev..

[4] Moore R.A., Derry S., Wiffen P.J., McQuay H.J. (2012). Amitriptyline for neuropathic pain and fibromyalgia in adults. Cochrane Database Syst. Rev..

[5] Häuser W., Urrútia G., Tort S., Üçeyler N., Walitt B. (2013). Serotonin and noradrenaline reuptake inhibitors (SNRIs) for fibromyalgia syndrome. Cochrane Database Syst. Rev..

[6] Üçeyler N., Sommer C., Walitt B., Häuser W. (2013). Anticonvulsants for fibromyalgia. Cochrane Database Syst. Rev..

[7] Macfarlane G.J., Kronisch C., Dean L.E., Atzeni F., Häuser W., Fluß E., Choy E., Kosek E., Amris K., Branco J. (2017). EULAR revised recommendations for the management of fibromyalgia. Ann. Rheum. Dis..

[8] Viganò A., Toscano M., Puledda F., Di Piero V. (2019). Treating Chronic Migraine with Neuromodulation: The Role of Neurophysiological Abnormalities and Maladaptive Plasticity. Front. Pharmacol..

[9] Conforto A.B., Amaro E., Gonçalves A.L., Mercante J.P., Guendler V.Z., Ferreira J.R., Kirschner C.C., Peres M.F. (2014). Randomized, proof-of-principle clinical trial of active transcranial magnetic stimulation in chronic migraine. Cephalalgia.

[10] O’Connell N.E., Marston L., Spencer S., DeSouza L.H., Wand B.M. (2018). Non-invasive brain stimulation techniques for chronic pain. Cochrane Database Syst. Rev..

[11] Fagerlund A.J., Hansen O.A., Aslaksen P.M. (2015). Transcranial direct current stimulation as a treatment for patients with fibromyalgia: A randomized controlled trial. Pain.

[12] Fregni F., Gimenes R., Valle A.C., Ferreira M.J., Rocha R.R., Natalle L., Bravo R., Rigonatti S.P., Freedman S.D., Nitsche M.A. (2006). A randomized, sham-controlled, proof of principle study of transcranial direct current stimulation for the treatment of pain in fibromyalgia. Arthritis Rheum..

[13] Villamar M.F., Wivatvongvana P., Patumanond J., Bikson M., Truong D.Q., Datta A., Fregni F. (2013). Focal modulation of the primary motor cortex in fibromyalgia using 4 × 1-ring high-definition transcranial direct current stimulation (HD-tDCS): Immediate and delayed analgesic effects of cathodal and anodal stimulation. J. Pain.

[14] Valle A., Roizenblatt S., Botte S., Zaghi S., Riberto M., Tufik S., Boggio P.S., Fregni F. (2009). Efficacy of anodal transcranial direct current stimulation (tDCS) for the treatment of fibromyalgia: Results of a randomized, sham-controlled longitudinal clinical trial. J. Pain Manag..

[15] Borckardt J.J., Bikson M., Frohman H., Reeves S.T., Datta A., Bansal V., Madan A., Barth K., George M.S. (2012). A pilot study of the tolerability and effects of high-definition transcranial direct current stimulation (HD-tDCS) on pain perception. J. Pain.

[16] Lefaucheur J.-P., Ayache S., Sorel M., Farhat W., Zouari H., de Andrade D.C., Ahdab R., Ménard-Lefaucheur I., Brugières P., Goujon C. (2012). Analgesic effects of repetitive transcranial magnetic stimulation of the motor cortex in neuropathic pain: Influence of theta burst stimulation priming. Eur. J. Pain.

[17] Huang Y.-Z., Edwards M.J., Rounis E., Bhatia K.P., Rothwell J.C. (2005). Theta burst stimulation of the human motor cortex. Neuron.

[18] Guerra A., Suppa A., Bologna M., D’Onofrio V., Bianchini E., Brown P., Di Lazzaro V., Berardelli A. (2018). Boosting the LTP-like plasticity effect of intermittent theta-burst stimulation using gamma transcranial alternating current stimulation. Brain Stimul..

[19] Antal A., Paulus W. (2013). Transcranial alternating current stimulation (tACS). Front. Hum. Neurosci..

[20] Villamar M.F., Volz M.S., Bikson M., Datta A., DaSilva A.F., Fregni F. (2013). Technique and considerations in the use of 4x1 ring high-definition transcranial direct current stimulation (HD-tDCS). J. Vis. Exp..

[21] Castillo-Saavedra L., Gebodh N., Bikson M., Diaz-Cruz C., Brandao R., Coutinho L., Truong D., Datta A., Shani-Hershkovich R., Weiss M. (2016). Clinically Effective Treatment of Fibromyalgia Pain With High-Definition Transcranial Direct Current Stimulation: Phase II Open-Label Dose Optimization. J. Pain.

[22] Bernardi L., Bertuccelli M., Formaggio E., Rubega M., Bosco G., Tenconi E., Cattelan M., Masiero S., Del Felice A. (2021). Beyond physiotherapy and pharmacological treatment for fibromyalgia syndrome: Tailored tACS as a new therapeutic tool. Eur. Arch. Psychiatry Clin. Neurosci..

[23] Wolfe F., Clauw D.J., Fitzcharles M.A., Goldenberg D.L., Häuser W., Katz R.L., Mease P.J., Russell A.S., Russell I.J., Walitt B. (2016). 2016 Revisions to the 2010/2011 fibromyalgia diagnostic criteria. Semin. Arthritis Rheum..

[24] Winer J.R., Mander B.A., Helfrich R.F., Maass A., Harrison T.M., Baker S.L., Knight R.T., Jagust W.J., Walker M.P. (2019). Sleep as a Potential Biomarker of Tau and β-Amyloid Burden in the Human Brain. J. Neurosci..

[25] Nguy B.-H.T., Liu W.-T., Chang Y.-T., Lin C.-P., Kang J.-H. (2020). Elevated tau and β-amyloid in the serum of fibromyalgia patients. CNS Spectr..

[26] Baptista A.F., Fernandes A.M.B., Sá K., Okano A.H., Brunoni A.R., Lara-Solares A., Iskandar A.J., Guerrero C., Amescua-García C., Kraychete D.C. (2019). Latin American and Caribbean consensus on noninvasive central nervous system neuromodulation for chronic pain management (LAC(2)-NIN-CP). Pain Rep..

[27] Desmeules J.A., Cedraschi C., Rapiti E., Baumgartner E., Finckh A., Cohen P., Dayer P., Vischer T.L. (2003). Neurophysiologic evidence for a central sensitization in patients with fibromyalgia. Arthritis Rheum..

[28] Polanía R., Paulus W., Nitsche M.A. (2012). Modulating cortico-striatal and thalamo-cortical functional connectivity with transcranial direct current stimulation. Hum. Brain Mapp..

[29] Lloyd D.M., Wittkopf P.G., Arendsen L.J., Jones A.K. (2020). Is Transcranial Direct Current Stimulation (tDCS) Effective for the Treatment of Pain in Fibromyalgia? A Systematic Review and Meta-Analysis. J. Pain.

[30] Khedr E.M., Omran E.A., Ismail N.M., El-Hammady D.H., Goma S.H., Kotb H., Galal H., Osman A.M., Farghaly H.S., Karim A.A. (2017). Effects of transcranial direct current stimulation on pain, mood and serum endorphin level in the treatment of fibromyalgia: A double blinded, randomized clinical trial. Brain Stimul..

[31] Roizenblatt S., Fregni F., Gimenez R., Wetzel T., Rigonatti S.P., Tufik S., Boggio P., Valle A.C. (2007). Site-specific effects of transcranial direct current stimulation on sleep and pain in fibromyalgia: A randomized, sham-controlled study. Pain Pract..

[32] Mendonca M.E., Simis M., Grecco L.C., Battistella L.R., Baptista A.F., Fregni F. (2016). Transcranial Direct Current Stimulation Combined with Aerobic Exercise to Optimize Analgesic Responses in Fibromyalgia: A Randomized Placebo-Controlled Clinical Trial. Front. Hum. Neurosci..

[33] Samartin-Veiga N., Pidal-Miranda M., González-Villar A.J., Bradley C., Garcia-Larrea L., O’Brien A.T., Carrillo-de-la-Peña M.T. (2021). Transcranial direct current stimulation of three cortical targets is no more effective than placebo as treatment for fibromyalgia: A double-blind sham-controlled clinical trial. Pain.

[34] Kold S., Graven-Nielsen T. (2021). Effect of anodal high-definition transcranial direct current stimulation on the pain sensitivity in a healthy population: A double-blind, sham-controlled study. Pain.

[35] Lim M., Kim J.S., Kim D.J., Chung C.K. (2016). Increased Low- and High-Frequency Oscillatory Activity in the Prefrontal Cortex of Fibromyalgia Patients. Front. Hum. Neurosci..

[36] Ahn S., Prim J.H., Alexander M.L., McCulloch K.L., Fröhlich F. (2019). Identifying and Engaging Neuronal Oscillations by Transcranial Alternating Current Stimulation in Patients With Chronic Low Back Pain: A Randomized, Crossover, Double-Blind, Sham-Controlled Pilot Study. J. Pain.

[37] May E.S., Hohn V.D., Nickel M., Tiemann L., Gil Ávila C., Heitmann H., Sauseng P., Ploner M. (2021). Modulating Brain Rhythms of Pain Using Transcranial Alternating Current Stimulation (tACS)—A Sham-Controlled Study in Healthy Human Participants. J. Pain.

[38] Arendsen L.J., Hugh-Jones S., Lloyd D.M. (2018). Transcranial Alternating Current Stimulation at Alpha Frequency Reduces Pain When the Intensity of Pain is Uncertain. J. Pain.

[39] Liu A., Vöröslakos M., Kronberg G., Henin S., Krause M.R., Huang Y., Opitz A., Mehta A., Pack C.C., Krekelberg B. (2018). Immediate neurophysiological effects of transcranial electrical stimulation. Nat. Commun..

[40] Neuling T., Rach S., Herrmann C.S. (2013). Orchestrating neuronal networks: Sustained after-effects of transcranial alternating current stimulation depend upon brain states. Front. Hum. Neurosci..

[41] Samartin-Veiga N., González-Villar A.J., Pidal-Miranda M., Vázquez-Millán A., Carrillo-De-La-Peña M.T. (2022). Active and sham transcranial direct current stimulation (tDCS) improved quality of life in female patients with fibromyalgia. Qual. Life Res..

[42] Kjær S.W., Rice A.S.C., Wartolowska K., Vase L. (2020). Neuromodulation: More than a placebo effect?. Pain.

[43] Colloca L. (2019). The Placebo Effect in Pain Therapies. Annu. Rev. Pharmacol. Toxicol..

[44] DosSantos M.F., Eferreira N., Toback R.L., Carvalho A.C., DaSilva A.F. (2016). Potential Mechanisms Supporting the Value of Motor Cortex Stimulation to Treat Chronic Pain Syndromes. Front. Neurosci..

[45] Vase L., Wartolowska K. (2019). Pain, Placebo, and Test of Treatment Efficacy: A Narrative Review. Br. J. Anaesth..

[46] Kortteenniemi A., Ortega-Alonso A., Javadi A.-H., Tolmunen T., Ali-Sisto T., Kotilainen T., Wikgren J., Karhunen L., Velagapudi V., Lehto S. (2020). Anodal tDCS Over the Left Prefrontal Cortex Does Not Cause Clinically Significant Changes in Circulating Metabolites. Front. Psychiatry.

[47] Desai P., Evans D., Dhana K., Aggarwal N.T., Wilson R.S., McAninch E., Rajan K.B. (2021). Longitudinal Association of Total Tau Concentrations and Physical Activity With Cognitive Decline in a Population Sample. JAMA Netw. Open.

[48] Reeves R.R., Ladner M.E. (2010). Antidepressant-induced suicidality: An update. CNS Neurosci Ther..

[49] Jick H., Kaye J.A., Jick S.S. (2004). Antidepressants and the risk of suicidal behaviors. JAMA.

[50] Wischnewski M., Compen B. (2022). Effects of theta transcranial alternating current stimulation (tACS) on exploration and exploitation during uncertain decision-making. Behav. Brain Res..

